# Characterizing Behavioral Engagement in Smartphone Cognitive Behavioral Therapy for Obsessive Compulsive Disorder: Secondary Analysis of a Randomized Controlled Trial

**DOI:** 10.2196/98129

**Published:** 2026-10-19

**Authors:** Hilary Weingarden, Geneva K Jonathan, Susanne S Hoeppner, Sara Velazquez, Ryan J Jacoby, Jennifer L Greenberg, Oliver Harrison, Sabine Wilhelm

**Affiliations:** 1 Department of Psychiatry, Massachusetts General Hospital and Harvard Medical School, 185 Cambridge St, Ste 2000, Boston, MA, 02114, United States, 1-617-726-6766; 2 PsychPlus, Houston, TX, United States

**Keywords:** obsessive compulsive disorder, OCD, cognitive behavioral therapy, CBT, engagement, smartphone, app, mHealth

## Abstract

**Background:**

Digital mental health interventions (DMHIs) are increasingly popular for their ability to disseminate evidence-based treatments in a scalable, accessible way. Engagement with digital treatments is critical for effectiveness but remains poorly defined and measured. Most studies rely on subjective self-reports or 1 or 2 objective metrics, overlooking the multidimensional nature of how users interact with DMHIs.

**Objective:**

This exploratory secondary analysis applied a road map for comprehensive behavioral engagement measurement, developed for a prior smartphone cognitive behavioral therapy program for body dysmorphic disorder, to a 12-week randomized controlled trial of smartphone cognitive behavioral therapy for obsessive compulsive disorder (OCD; N=60). Aims were to (1) comprehensively characterize behavioral engagement using 21 objective app analytics-based variables, (2) identify distinct behavioral engagement profiles via cluster analysis, (3) assess whether profiles were differentially associated with OCD symptom change, and (4) evaluate associations between profiles and pretreatment credibility, expectancy, posttreatment satisfaction, and perceived app quality.

**Methods:**

Behavioral engagement was measured via 21 objective variables derived from app analytics, spanning use volume, frequency, feature usage, session duration, and temporal patterns. Hierarchical agglomerative cluster analysis identified engagement profiles. Associations between cluster membership and OCD severity change (Yale-Brown Obsessive Compulsive Scale) were examined using hierarchical generalized linear mixed models. Objective measures were complemented with subjective self-reports: namely, cluster differences in treatment credibility, expectancy, satisfaction, and perceived app quality were examined using ANOVAs and Wilcoxon rank-sum tests.

**Results:**

Cluster analysis indicated a 3-cluster solution representing high (cluster 1: 21/60, 35%), moderate (cluster 2: 31/60, 52%), and low (cluster 3: 8/60, 13%) app users. All 3 clusters experienced significant reductions in OCD severity, with no significant differences in pre- to posttreatment Yale-Brown Obsessive Compulsive Scale change across clusters. Descriptively, cluster 1 had a higher rate of treatment responders than cluster 3. While clusters 1 and 2 had similar perceptions of treatment credibility and expectancy at baseline and satisfaction at posttreatment, cluster 3 perceived the intervention as significantly less credible, had lower expectations, and reported significantly lower satisfaction than cluster 2. All clusters rated app quality as similarly high.

**Conclusions:**

Comprehensive behavioral engagement measurement is feasible and informative across different DMHIs and clinical populations. The finding that all engagement profiles showed comparable symptom improvement challenges simple dose-response assumptions and may suggest that multiple paths of engagement can be therapeutically beneficial; nonsignificant differences in improvement across clusters may also reflect limited power to detect differences. Pretreatment credibility and expectancy are associated with engagement patterns, identifying a potential modifiable intervention target.

## Introduction

Digital mental health interventions (DMHIs) have the potential to expand access to care, offering scalable and flexible treatment options. However, their effectiveness depends on user engagement, a critical yet commonly overlooked factor in digital tools [1-3]. Engagement is a multidimensional construct that encompasses behavioral indicators of intervention use (eg, frequency, duration, breadth, and depth) and subjective experience (eg, affective response, interest, and attentional focus) during interactions with the intervention [3,4]. Despite its importance, engagement measurement has historically been oversimplified: most studies use self-report measures of satisfaction or acceptability that are vulnerable to demand characteristics [5,6]; those that incorporate objective data typically include only 1 or 2 such variables, such as login counts or total time on the app [6-8].

Considering only 1 or 2 objective measures of engagement overlooks nuances of how users interact with DMHIs. For example, 2 users may log into an app the same number of times, but one may do so consistently over 8 weeks, whereas the other logs in intensively at first and then disengages. Certain intervention content, such as exposure with response prevention (ERP) for obsessive compulsive disorder (OCD), may be most effective when practiced consistently over time. Thus, these 2 usage patterns reflect meaningful differences in treatment exposure, yet they appear identical when measured by login counts alone. Similarly, measuring total time on an app does not capture *how* time is distributed across content areas. Time spent on ERP modules may be more strongly associated with OCD symptom improvement than time spent on less therapeutically active modules. Altogether, there is value in measuring engagement comprehensively to capture quantity, quality, and context of use.

We previously developed a road map for comprehensive behavioral engagement measurement in a trial of smartphone-delivered cognitive behavioral therapy (CBT) for body dysmorphic disorder (BDD) [9]. Using 19 app analytics-derived variables ranging from use volume, frequency, feature usage, session duration, and temporal regularity, cluster analysis identified three engagement profiles: (1) “deep” users, who engaged selectively with content but for extended durations; (2) “samplers,” who interacted briefly with most content; and (3) “light” users, with minimal and sporadic use. Engagement profiles were not significantly associated with differential treatment outcomes, although trends suggested that deep engagement may be linked to greater improvement compared with light use. This work demonstrated a novel approach to comprehensive behavioral engagement measurement.

Our initial study in smartphone CBT for BDD was limited to a single clinical condition and intervention. However, each DMHI has unique features, content, and capabilities that will drive different use patterns, and each clinical target may require different patterns of use to achieve adequate treatment response. Thus, a next step is to test whether this measurement approach adapts and generalizes to new populations and interventions. Digital CBT for OCD offers a relevant second test of this measurement approach for a couple of reasons. First, access to evidence-based treatment for OCD is limited by a shortage of providers [10]. Many who do initiate OCD treatment do not receive guideline-concordant ERP [11,12]. DMHIs may help address these barriers, but their success hinges on user engagement. Second, CBT for OCD presents unique engagement challenges that give an opportunity to test whether our approach can be tailored to the distinctive considerations of a specific condition and intervention. Namely, ERP is aversive and may elicit disengagement [13], particularly in self-directed DMHIs, where no therapist is present to provide support and accountability. Altogether, digital CBT for OCD offers a relevant test case to evaluate whether our behavioral engagement measurement approach can capture clinically meaningful patterns of use in a distinct condition and intervention.

Our prior work measuring engagement in smartphone CBT for BDD was also limited by an exclusive focus on capturing engagement through app analytics; we did not consider subjective engagement dimensions in tandem. For the most comprehensive view into user engagement patterns, objective behavioral metrics can be complemented and supplemented by participant-rated indices of engagement, such as treatment credibility, expectancy, and satisfaction, which reflect user attitudes about the intervention. For instance, a treatment’s early credibility, and participants’ initial expectancies about treatment, may shape behavioral engagement. Prior research suggests that treatment credibility and expectancy predict outcomes in exposure-based treatment for OCD [14,15]. However, to our knowledge, whether self-reported credibility and expectancy predict patterns of objective behavioral engagement with a DMHI has not been examined. Establishing such connections would provide actionable targets for improving intervention uptake and sustained use.

In this study, we applied and extended our prior behavioral engagement measurement road map [9] to examine objective (app analytics) and subjective (self-report) measures of engagement with smartphone-delivered CBT for OCD (“*Perspectives OCD*,” N=60) [16]. This study extends our prior work in 3 ways: by testing the comprehensive behavioral engagement measurement approach in a new clinical population and intervention, by integrating objective and subjective engagement indicators to address a limitation of the prior study, and by examining whether pretreatment credibility and expectancy predict objective engagement profiles—a novel question in the DMHI literature. Aims were to (1) comprehensively characterize behavioral engagement; (2) identify distinct behavioral engagement profiles among users, via cluster analysis of 21 variables derived from app analytics; (3) assess whether engagement profiles were associated with symptom change over time; and (4) evaluate associations between engagement profiles and subjective indicators of treatment credibility, expectancy, satisfaction, and perceived app quality.

Based on our prior findings in BDD [9] and broader DMHI research [17,18], we expected to identify distinct behavioral engagement profiles across multiple dimensions. While we hypothesized that profiles characterized by greater engagement, particularly involving more interaction with ERP content, would be associated with greater clinical improvement and more favorable ratings on measures of treatment credibility, expectancy, satisfaction, and quality, we expected these associations to be modest based on mixed findings in extant literature.

## Methods

### Overview and Study Design

This is a secondary analysis of a 12-week randomized controlled trial comparing Perspectives OCD to HealthWatch, an online general health psychoeducation program validated as an attentional control for DMHIs [19]. In this study, only data from the Perspectives OCD condition were used (N=60). Full descriptions of study design, intervention, eligibility criteria, and procedures are reported in the primary manuscript [16] and clinicaltrials.gov record (NCT04136626). A STROBE (Strengthening the Reporting of Observational Studies in Epidemiology) checklist is provided in Multimedia Appendix 1 [20].

### Smartphone CBT for OCD

Perspectives OCD is a self-paced Android and iOS-compatible app with seven core modules that cover (1) psychoeducation, which oriented users to the CBT model of OCD and guided personalized goal setting; (2) cognitive restructuring to identify and reappraise OCD-related cognitive distortions; (3) ERP, which guided users through graded exposure to OCD triggers while refraining from compulsions; (4) mindfulness and mindful grounding (2 modules), which included audio exercises teaching mindfulness and attentional retraining skills; (5) values-based living and self-esteem, which helped users set activity goals aligned with their values and identify and modify negative core beliefs; and (6) relapse prevention. During goal setting, participants selected 1-4 OCD symptom domains (ie, contamination, over responsibility, perfectionism, aggressive, sexual, and scrupulosity obsessions). Within the ERP modules, the app then presented symptom-specific graded exposure hierarchies for each selected domain, which participants could complete in any order, skip if irrelevant, and supplement with user-created custom exposures. All app modules unlocked sequentially and contained psychoeducational reading complemented by short interactive exercises that could be repeated as often as desired. Useful exercises could be added to “favorites” for easy repetition.

Perspectives OCD was delivered with light-touch coach support. Each participant was assigned a trained, bachelor’s-level coach who provided 2 brief phone calls at onboarding and midtreatment, respectively, as well as asynchronous in-app messaging. Coach support was focused on encouraging regular use, clarifying CBT content, and troubleshooting practical barriers, without delivering psychotherapy outside the app. Participants were encouraged to use the app daily and progress at their own pace over 12 weeks. See Wilhelm et al [16] for a full description of the intervention and participant journey.

### Participants

Eligible participants included adults aged 18 years and older in the United States with a primary diagnosis of OCD, confirmed via the Mini International Neuropsychiatric Interview (MINI; [21]), who owned a smartphone with a data plan. Individuals were excluded if they were in concurrent psychotherapy or used another OCD-related app, had received 4 or more prior sessions of CBT for OCD, or had initiated or changed psychotropic medication in the past 2 months; stable medication was permitted. Additional exclusion criteria included current severe substance use disorder, current severe major depressive disorder, current posttraumatic stress disorder, lifetime bipolar disorder or psychosis, and acute suicidal ideation. Participants were recruited nationally from the Massachusetts General Hospital Center for OCD and Related Disorders via social media advertisements, professional organizations, and colleague referrals, between July 2021 and July 2023.

### Procedures

#### Ethical Considerations

Procedures were approved by the Mass General Brigham Institutional Review Board (protocol number 2018P001669) and monitored by a Data Safety and Monitoring Board. The primary study’s informed consent form covered use of deidentified data for secondary research analyses. After providing written informed consent, participants were randomized 1:1 to Perspectives OCD or HealthWatch, using computer-generated block randomization (block size 4), stratified by stable psychotropic medication status. The randomized assignment was concealed from participants until baseline clinician-rated assessments were completed and eligibility was confirmed; all participants were provided onboarding instructions to their assigned treatment the same day. As noted previously, only those participants assigned to the Perspectives OCD condition were included in current analyses. Data were stored deidentified on the secure REDCap (Vanderbilt University) [22,23] and Dropbox for Business platforms to protect participant privacy. Participants were compensated US $25 for midtreatment and posttreatment assessments.

#### Assessments

Participants completed a screening and baseline assessment that included diagnostic interviews, eligibility checks, and baseline demographics and clinical measures. Clinician-rated measures were conducted via videoconference at baseline, midtreatment (week 6), and posttreatment (week 12) by blinded doctoral-level independent evaluators (IEs). Self-reports were completed electronically through REDCap [22,23].

### Measures

#### Diagnostic and Outcome Measures

At baseline, the MINI [21] and Columbia Suicide Severity Rating Scale [24] were administered by trained IEs to assess eligibility and characterize the sample. The MINI is a brief, well-validated structured psychiatric diagnostic assessment that evaluates presence or absence of mental illnesses using the *DSM-5-TR* (*Diagnostic and Statistical Manual of Mental Disorders* [Fifth Edition, Text Revision]) criteria [25]. The Columbia Suicide Severity Rating Scale is a widely used and well-validated, clinician-administered assessment of severity of suicide ideation and imminence of current and past suicide risk. The primary trial outcome was OCD severity, assessed with the 10-item clinician-administered Yale-Brown Obsessive Compulsive Scale (Y-BOCS [26]). Total Y-BOCS scores can range from 0 to 40; higher scores indicate greater severity. Severity ranges derived from Y-BOCS total scores include mild (0‐13), moderate (14-25), moderate-severe (26-34), and severe (35-40) categories [27]. Internal consistency was 0.74 at baseline and 0.91 at the end of treatment in this sample. IEs also assessed improvement using the Clinical Global Impressions–Improvement scale (CGI-I; [28]), a clinician-rated item measuring the extent of illness improvement, ranging from 1 (“very much improved”) to 7 (“very much worse”). Treatment response was defined as a three-category variable [29]: (1) full response: ≥35% reduction in Y-BOCS at week 12 plus a CGI-I rating of 1 (“very much improved”) or 2 (“much improved”), (2) partial response: <35% but ≥25% reduction in Y-BOCS plus a CGI-I ≤3 (at least “minimally improved”), and (3) no response: <25% reduction in Y-BOCS and/or a CGI-I rating >3 (no change or OCD symptom worsening). Remission was defined as a Y-BOCS total score ≤12 at week 12 [29]. Study dropouts (3/60, 5%) were counted as nonresponders and not in remission.

#### App Engagement

App engagement was described through 21 objective variables, belonging to five categories derived from participant actions logged passively by the app, from onboarding until the end of treatment:

1. Amount of use: These variables included (1) total minutes interacting with the app, (2) total number of sessions (ie, times the participant returned to the app), and (3) total number of days the participant used the app. We defined an app session as a sequence of user-initiated actions within the app separated by <5 minutes between events [30]. When the app had content (audio, video, or ERP tasks) that lasted >5 minutes, use was counted as continued activity within the same session. To reduce the impact of session time outliers (from missing log entries of app closures or pauses), outliers approximately >99th percentile of times observed for that app action were capped at the maximum time expected for each app action (eg, 5 minutes for creating a checklist item; 30 minutes for completing a long ERP exercise); this affected 0.14% (53/36,564) of app actions.
2. Frequency and inactivity periods: These variables included (1) app use frequency (log-transformed mean time, in minutes, between consecutive app sessions) and (2) inactivity periods (number of times a participant was inactive for 7+ consecutive days before returning to the app).
3. Use of key app features: These variables included (1) percentage of main modules completed, (2) percentage of main exercises completed, (3) percentage of exercises completed out of those started by the participant, (4) percentage of ERP exercises completed out of those started, (5) number of ERP exercises completed, (6) number of custom ERP exercises created, and (7) number of chat messages sent to the coach. Main modules included the 7 core modules assigned to all participants, plus the first symptom-matched ERP hierarchy that the participant selected. Main exercises were all exercises within the main modules. For the number of ERP exercises completed per person, 5 outliers (range 33-85) were recoded to a maximum of 30 before analysis (affecting 5/60, 8%) of participant-level app use summary data.
4. Duration of sessions: App sessions were categorized into 4 bins based on duration: 10 seconds to 3 minutes (bin 1), >3-5 minutes (bin 2), >5-10 minutes (bin 3), and >10 minutes (bin 4). Sessions <10 seconds were judged to be too short for meaningful engagement and were excluded (affecting 778/4317, 18.0%). App use duration variables included (1) percentages of sessions in each of the 4 duration bins, (2) session duration entropy (ie, diversity of session lengths), and (3) participants’ median session duration (in minutes). Session duration entropy was calculated as Shannon entropy, where H = ∑^4^_n=1_ (proportion_sessions_bin_n_ * log(proportion_sessions_bin_n_)).
5. Consistency of use over time: These variables included percentage of total use time during weeks 1‐4, weeks 5‐8, and weeks 9‐12, respectively.

#### Self-Reported Treatment Usage, Credibility, Expectancy, Satisfaction, and Perceived Quality

Because analytics-derived variables cannot capture skills practice that occurs off the app, we administered self-report items at weeks 6 (midtreatment) and 12 (posttreatment), asking participants to estimate total time (in minutes) spent practicing treatment skills per week, and the proportions spent doing so on versus off the app, to calculate minutes spent practicing skills on and off the app. Data on this measure were impacted by dropout (3/60, 5%), missed assessments or skipped questions on this measure (7/60, 12%), and data that could not be reconciled into percentages that added up to 100% (9/60, 15%), leading to missing estimates for 32% (19/60) of participants.

Treatment credibility (3 items) and expectancy (3 items) were assessed at baseline after randomization using the Credibility and Expectancy Questionnaire [31]. Subscale scores can range from 3 to 27, with higher scores indicating greater treatment credibility or expectancy. The credibility subscale had an internal consistency of α=.76, and the expectancy subscale had an internal consistency of α=.87. A sample credibility item is “How logical does the type of treatment offered to you seem?” and a sample expectancy item is “How much improvement in your OCD do you think will occur?”

Client satisfaction was assessed posttreatment using the 8-item Client Satisfaction Questionnaire (CSQ-8; [32]). Total scores can range from 8 to 32, with higher scores indicating greater satisfaction. The CSQ-8 had an internal consistency of α=.95 in this sample. A sample item is “How would you rate the quality of service you received?” At posttreatment, participants were also asked to rate the app’s quality using the 26-item user version of the Mobile Application Rating Scale (uMARS [33]). Sixteen items evaluate an app’s quality with respect to engagement, functionality, aesthetics, and information via an overall mean score ranging from 1 to 5, where higher scores indicate better quality. Internal consistency of the uMARS was α=.81. A supplementary item asks, “Would you recommend this app to people who might benefit from it?” with answer options ranging from 1 (“Not at all. I would not recommend this app to anyone”) to 5 (“Definitely I would recommend this app to everyone”). For both the CSQ-8 and the uMARS scales, 12% (7/60) of participants had missing data (3 dropouts, 4 people with skipped survey responses) and were excluded from data analysis for these outcomes.

### Statistical Analysis

The present analyses were not preregistered and are exploratory. Differences were evaluated at α=.05, and analyses were conducted in SAS (version 9.4; SAS Institute) for Windows. All participants downloaded the app and initiated app use, so app use variables were calculated for all.

#### Aim 1: Describe App Use Across the Sample

Overall patterns of behavioral engagement were summarized descriptively.

#### Aim 2: Determine Clusters of App Users

To identify subgroups with differing patterns of app use, we used hierarchical agglomerative cluster analysis using Ward’s minimum variance method based on squared Euclidean distance. Individuals with the least degree of difference in clustering variables are joined. Data points or clusters are then continuously joined, one at a time, with the goal of minimizing within-cluster sums of squares, until a single cluster remains containing all participants. For interpretation, a cut point is chosen at which clusters that are “distant” appear meaningfully different. We used an inverse scree test based on semipartial *R*^2^ and inspection of the dendrogram to select the optimal number of clusters to retain, balanced by the need to maintain clusters of sufficient size. A heatmap of variable correlations, scree plot, and bootstrapped dendrogram are available in Multimedia Appendices 2–4, respectively. Percentages were converted to proportions to fall between 0 and 1, and other variables were scaled using the minimum-maximum scaler to bring their values between 0 and 1, while preserving their distribution. To examine which app use characteristics explained cluster differences, we used 1‐way ANOVAs for normally distributed variables and Wilcoxon rank-sum tests for strongly skewed variables. Cluster group membership served as the only independent variable.

#### Aim 3: Examine Y-BOCS Change Over Time by App Use Cluster

We used a hierarchical generalized linear mixed model with repeated measures to model Y-BOCS total scores in each cluster over time. The model used time (baseline, week 6, and week 12), cluster group, time-by-cluster interaction, stratification (yes/no baseline medication use), and stratification group-by-time interaction as fixed effects. Time was modeled as a repeated measure using a Toeplitz covariance structure with heterogeneous variances. The model used all available data, handling missing data via maximum likelihood estimation. We used specific contrasts to estimate differences in change from baseline to end-of-treatment between clusters. Due to the exploratory nature of analyses and small sample sizes within clusters, group differences were not adjusted for multiple comparisons. Results are presented as model-adjusted least-squares mean (LSM) with 95% confidence limits. Treatment response, a categorical recoding of Y-BOCS percent change, is reported descriptively only; the 3 treatment dropouts were coded as nonresponders.

#### Aim 4: Examine Cluster Differences in Self-Reported Treatment Usage, Credibility, Expectancy, Satisfaction, and App Quality

Cluster differences in self-reported treatment usage, credibility, expectancy, satisfaction, and app quality were examined using 1‐way ANOVAs for normally distributed variables (most outcomes) and Wilcoxon rank-sum tests for strongly skewed variables (ie, total time practicing skills on and off the app). Cluster group membership served as the only independent variable.

Effect sizes (ESs) were calculated as Hedges *g*, a measure similar to Cohen’s *d* with a correction for unequal sample sizes, where ESs of ±0.2, ±0.5, and ±0.8 represent small, medium, and large ESs, respectively. For Tables 1 and 2, pairwise comparison *P* values are presented as raw *P* values; *P* values adjusted for false discovery rate (FDR [34]), as well as details of the statistical tests, are available in Table S1 in Multimedia Appendix 5 and Table S1 in Multimedia Appendix 6. *P* values were adjusted for all other *P* values in the same table. Any *P* value still significant after FDR adjustment are marked with a footnote in Tables 1 and 2.

**Table 1.** App use characteristics of participants (N=60) in a trial studying the efficacy of a 12-week smartphone cognitive behavioral therapy program for obsessive-compulsive disorder, by app use cluster.

App use variables	Cluster 1 (n=21), mean (SD) or median (IQR)	Cluster 2 (n=31), mean (SD) or median (IQR)	Cluster 3 (n=8), mean (SD) or median (IQR)	Cluster 1 vs cluster 2		Cluster 2 vs cluster 3		Cluster 1 vs cluster 3	
				*P* value	ESs^a^	*P* value	ESs	*P* value	ESs
Amount (volume) of use									
Total app use (minutes), median (IQR)	740.7 (606.4, 897.6)	245.7 (158.6, 325.2)	166.3 (58.2, 224.9)	<.001^b^	N/A^c^	.02^b^	N/A	<.001^b^	N/A
Total number of app sessions, mean (SD)	91.1 (34.3)	44.1 (13.7)	32.3 (19.7)	<.001^b^	1.95	.21	0.79	<.001^b^	1.89
Total number of days of app use, mean (SD)	51.0 (16.2)	31.2 (10.5)	22.0 (12.5)	<.001^b^	1.51	.08	0.84	<.001^b^	1.89
Frequency and inactivity periods									
App use frequency (inactivity), log-min^d^, mean (SD)	7.2 (0.3)	7.9 (0.3)	8.4 (1.0)	<.001^b^	−2.33	.01^b^	−0.98	<.001^b^	−2.10
Number of inactivity periods of 7+ days, median (IQR)	0.0 (0.0, 0.0)	2.0 (0.0, 3.0)	1.5 (1.0, 3.0)	<.001^b^	N/A	.63	N/A	<.001^b^	N/A
Use of key app features									
Percentage of main modules completed, median (IQR)	87.5 (75.0, 100.0)	50.0 (50.0, 62.5)	37.5 (37.5, 43.8)	<.001^b^	N/A	.007^b^	N/A	<.001^b^	N/A
Percentage of main exercises completed, median (IQR)	96.4 (82.1, 100.0)	64.3 (60.7, 78.6)	48.2 (42.2, 51.8)	<.001^b^	N/A	<.001^b^	N/A	<.001^b^	N/A
Percentage of all exercises (started) completed, median (IQR)	96.4 (93.4, 99.2)	92.3 (85.7, 100.0)	66.8 (54.6, 82.1)	.12	N/A	.007^b^	N/A	.002^b^	N/A
Percentage of ERP^e^ exercises (started) completed, median (IQR)	97.1 (88.2, 100.0)	90.0 (75.0, 100.0)	5.0 (0.0, 35.0)	.11	N/A	<.001^b^	N/A	<.001^b^	N/A
Number of ERP exercises completed, median (IQR)	18.0 (16.0, 24.0)	7.0 (4.0, 10.0)	0.5 (0.0, 1.5)	<.001^b^	N/A	<.001^b^	N/A	<.001^b^	N/A
Number of custom ERP exercises created, median (IQR)	0.0 (0.0, 1.0)	0.0 (0.0, 0.0)	0.0 (0.0, 0.0)	.21	N/A	.43	N/A	.15	N/A
Number messages sent to coach, mean (SD)	19.7 (11.2)	10.4 (6.2)	6.5 (6.6)	<.001^b^	1.09	.24	0.62	<.001^b^	1.29
Duration of sessions									
Percentage of sessions >10 seconds to 3 minutes, mean (SD)	46.3 (13.5)	57.5 (15.0)	66.7 (7.3)	.006^b^	−0.78	.10	−0.66	<.001^b^	−1.67
Percentage of sessions >3 minutes to 5 minutes, mean (SD)	9.1 (3.8)	10.3 (5.2)	7.4 (6.8)	.36	−0.26	.15	0.52	.44	0.36
Percentage of sessions >5 minutes to 10 minutes, mean (SD)	12.5 (4.7)	12.4 (5.8)	13.0 (8.9)	.95	0.00	.81	−0.08	.85	−0.08
Percentage of sessions >10 minutes, mean (SD)	32.1 (13.4)	19.8 (13.1)	12.9 (8.0)	.001^b^	0.93	.18	0.56	<.001^b^	1.57
Entropy of session duration, median (IQR)	1.6 (1.6, 1.7)	1.6 (1.4, 1.7)	1.3 (1.1, 1.5)	.16	N/A	.04	N/A	<.001^b^	N/A
Median session duration, minutes, median (IQR)	3.0 (2.3, 5.9)	2.2 (1.5, 3.5)	1.6 (1.0, 2.1)	.01^b^	N/A	.07	N/A	<.001^b^	N/A
Consistency of use over time									
Percentage of app minutes in weeks 1‐4, mean (SD)	49.1 (17.9)	60.9 (19.6)	78.0 (16.6)	.03^b^	−0.62	.02^b^	−0.90	<.001^b^	−1.65
Percentage of app minutes in weeks 5‐8, mean (SD)	26.2 (11.8)	24.5 (14.9)	15.0 (13.4)	.65	0.12	.09	0.65	.05	0.92
Percentage of app minutes in weeks 9‐12, mean (SD)	24.7 (15.6)	14.7 (10.8)	7.0 (8.0)	.006^b^	0.77	*.*12	0.75	.001^b^	1.26

a ESs: effect sizes, calculated as Hedges *g* in SD units.

b *P* value is still significant at *P*<.05 after false discovery rate adjustment; for details, see Table S1 in Multimedia Appendix 5.

c N/A: not applicable.

d log-min: log-transformed number of minutes.

e ERP: exposure with response prevention.

**Table 2.** Self-reported treatment usage, credibility, expectancy, and satisfaction by participants in 12-week smartphone cognitive behavioral therapy program for obsessive-compulsive disorder in each identified app use cluster^a^.

	Cluster 1 (n=21)	Cluster 2 (n=31)	Cluster 3 (n=8)	Pairwise comparisons					
				Cluster 1 vs cluster 2		Cluster 2 vs cluster 3		Cluster 1 vs cluster 3	
				*P* value	ESs^b^	*P* value	ESs	*P* value	ESs
Treatment usage									
Estimated app use time (minutes), median (IQR)	441.0 (270.0, 600.0)	240.0 (132.0, 360.0)	102.6 (6.0, 378.0)	*.01* ^c^	N/A^d^	N/A	N/A	N/A	N/A
Estimated skill use time outside app (minutes), median (IQR)	526.5 (365.4, 1071.0)	477.0 (288.8, 846.0)	167.4 (54.0, 326.0)	.45	N/A	N/A	N/A	N/A	N/A
Credibility and expectancy (CEQ^e^)									
Credibility, mean (SD)	18.4 (3.5)	20.4 (3.7)	15.9 (3.6)	.06	−0.55	*.003* ^*f*^	1.22	.09	0.71
Expectancy, mean (SD)	16.2 (4.8)	17.5 (4.6)	12.8 (5.7)	.35	−0.28	*.02* ^c^	0.97	.10	0.67
Satisfaction									
CSQ-8^g^ total, mean (SD)	25.5 (5.3)	26.6 (4.1)	21.0 (7.1)	.46	−0.24	*.02* ^c^	1.16	.07	0.77
uMARS^h^ total, mean (SD)	4.1 (0.5)	4.1 (0.4)	4.2 (0.4)	.91	0.00	.69	−0.25	.65	−0.21
uMARS recommend app, mean (SD)	3.7 (0.9)	4.1 (0.8)	3.2 (1.6)	.19	−0.48	.06	0.90	.27	0.45

a Due to missing data, pairwise comparisons with cluster 3 for treatment usage were not feasible.

b ESs: effect sizes, calculated as Hedges *g* in SD units.

c Raw *P* values shown, where italicized values indicate raw *P*<.05.

d N/A: not applicable.

e CEQ: Credibility Expectancy Questionnaire.

f *P* value is still significant at *P*<.05 after false discovery rate adjustment.

g CSQ-8: 8-item Client Satisfaction Questionnaire.

h uMARS: user version of the Mobile Application Rating Scale.

## Results

### Sample Description

#### Overview

All participants had some app use data and were included in analyses. Sample demographics have been described previously [16]. Briefly, participants were on average aged 39.0 (SD 14.7) years. Overall, 78% (47/60) identified as women and 17% (10/60) as men, while 5% (3/60) identified as transgender, nonbinary, or questioning. The majority (48/60, 80%) identified as White; 5% (3/60) identified as Hispanic or Latino. The mean Y-BOCS total score at baseline was 25.1 (SD 4.2), with 53% (32/60) of participants reporting symptoms in the moderate range, 45% (27/60) in the moderate-severe range, and 2% (1/60) in the severe range.

#### Aim 1: Describe App Use Across the Sample

Participants’ app use across the 21 objective variables is shown in Table 1 (for overall sample descriptives, see Table S1 in Multimedia Appendix 7), and participants’ minutes of app use are displayed in Figure 1. Overall, participants spent substantial time on the app and returned frequently. Participants used the app most during the first 4 weeks, although app use was well sustained over time, with little drop-off in active app users (ie, participants who used the app at least once a week) from week 1 (60/60, 100%) to week 12 (52/60, 86%). The lowest number of active app users was observed in week 10 (51/60, 85%). Overall, 68% (41/60) of participants logged at least 1 session weekly for the entire treatment period. The participant with the lowest app use had 12.9 minutes of app use across 3 separate days.

**Figure 1.**
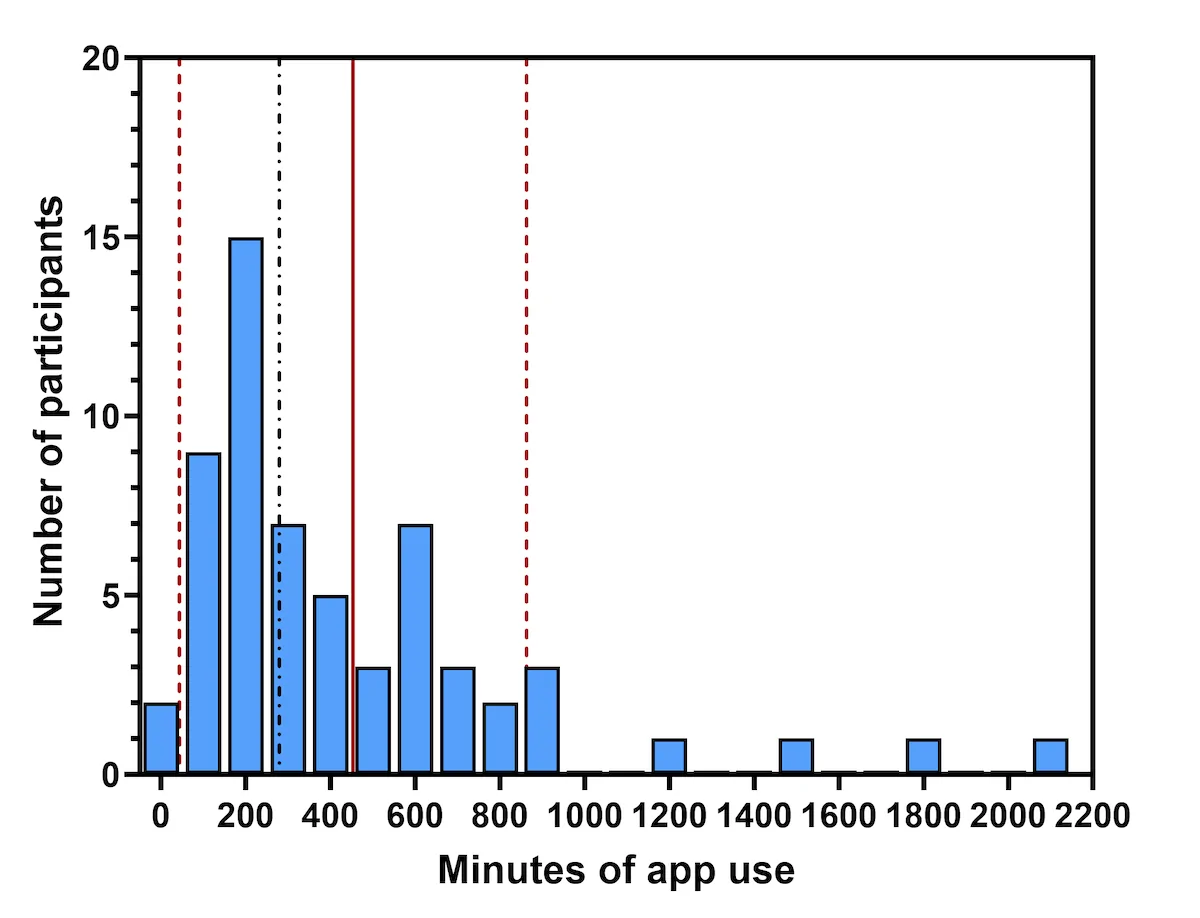
Histogram of participants’ total app use (in minutes) during the 12-week treatment period with a smartphone cognitive behavioral therapy program for obsessive-compulsive disorder. The median is shown in a black dashed line, the mean is shown in a solid red line, and mean +/- 1 SD is shown in dashed red lines.

#### Aim 2: Determine Clusters of App Users

Cluster analysis indicated a 3-cluster solution representing distinct groups of high (cluster 1: 21/60, 35% of users), moderate (cluster 2: 31/60, 52%), and low (cluster 3: 8/60, 13%) app use across dimensions. Table 1 shows app use variables by cluster (see Table S1 in Multimedia Appendix 7 for overall descriptives and Table S1 in Multimedia Appendix 5 for statistical detail). Cluster 1 spent more than twice as much time on the app compared with cluster 2, which in turn spent significantly more time on the app than cluster 3. Cluster 1 users also completed the most exercises (including ERP), sent the most chat messages to coaches, had more sessions of 10+ minutes, and were more active in the final weeks of treatment. While cluster 3 spent substantial time on the app, they completed less than half of app content (both modules and exercises) and completed few to no ERP exercises. Cluster 3 tended to use the app most in weeks 1‐4, with the percentage of use time falling off sharply thereafter. All but one of the pairwise differences between clusters (comparison of cluster 2 vs cluster 3 in entropy of session duration) were still significant after FDR adjustment (Table 1; Table S1 in Multimedia Appendix 5).

#### Aim 3: Examine Y-BOCS Change Over Time by App Use Cluster

Overall, participants’ Y-BOCS scores decreased significantly from baseline (LSM 24.9, 95% CI 23.5-26.2) to week 12 (16.4, 14.2-18.5); the difference was 8.5 (6.2-10.9; *P*<.001). However, cluster groups did not differ in pre- to posttreatment Y-BOCS change: the difference in cluster 1 vs cluster 2 pre- to postchange was 0.6 (−3.5 to 4.7; *t*_54.3_=0.30, *P*=.77; posttreatment ESs: −0.25 SD units); for cluster 2 vs cluster 3, the difference was 1.7 (−4.4 to 7.9; *t*_59.2_=0.56, *P*=.58; posttreatment ESs: 0.05 SD units); and for cluster 1 vs cluster 3, the difference was 2.3 (−4.2 to 8.9; *t*_58.7_=0.72, *P*=.48; posttreatment ESs: −0.21 SD units; Figure 2). At baseline, 52% (11/21), 48% (15/31), and 75% (6/8) of participants reported symptoms in the moderate range in clusters 1, 2, and 3, respectively; 48% (10/21), 48% (15/31), and 25% (2/8) of participants reported symptoms in the moderate-severe category; and only 3% (1/31) reported severe symptoms in cluster 2. We were unable to detect significant differences in Y-BOCS scores between clusters at baseline (cluster 1: LSM 24.9, 95% CI 22.8-27.0, cluster 2: 25.7, 24.1-27.4, and cluster 3: 24.0, 20.8-27.2; pairwise differences: cluster 1 vs cluster 2: *t*_55.1_=−0.67, *P*=.50, ESs: 0.25 SD units; cluster 1 vs cluster 3: *t*_55.1_=0.46, *P*=.65, ESs: −0.15 SD units; and cluster 2 vs cluster 3: *t*_55.1_=0.98, *P*=.33, ESs: −0.40 SD units).

**Figure 2.**
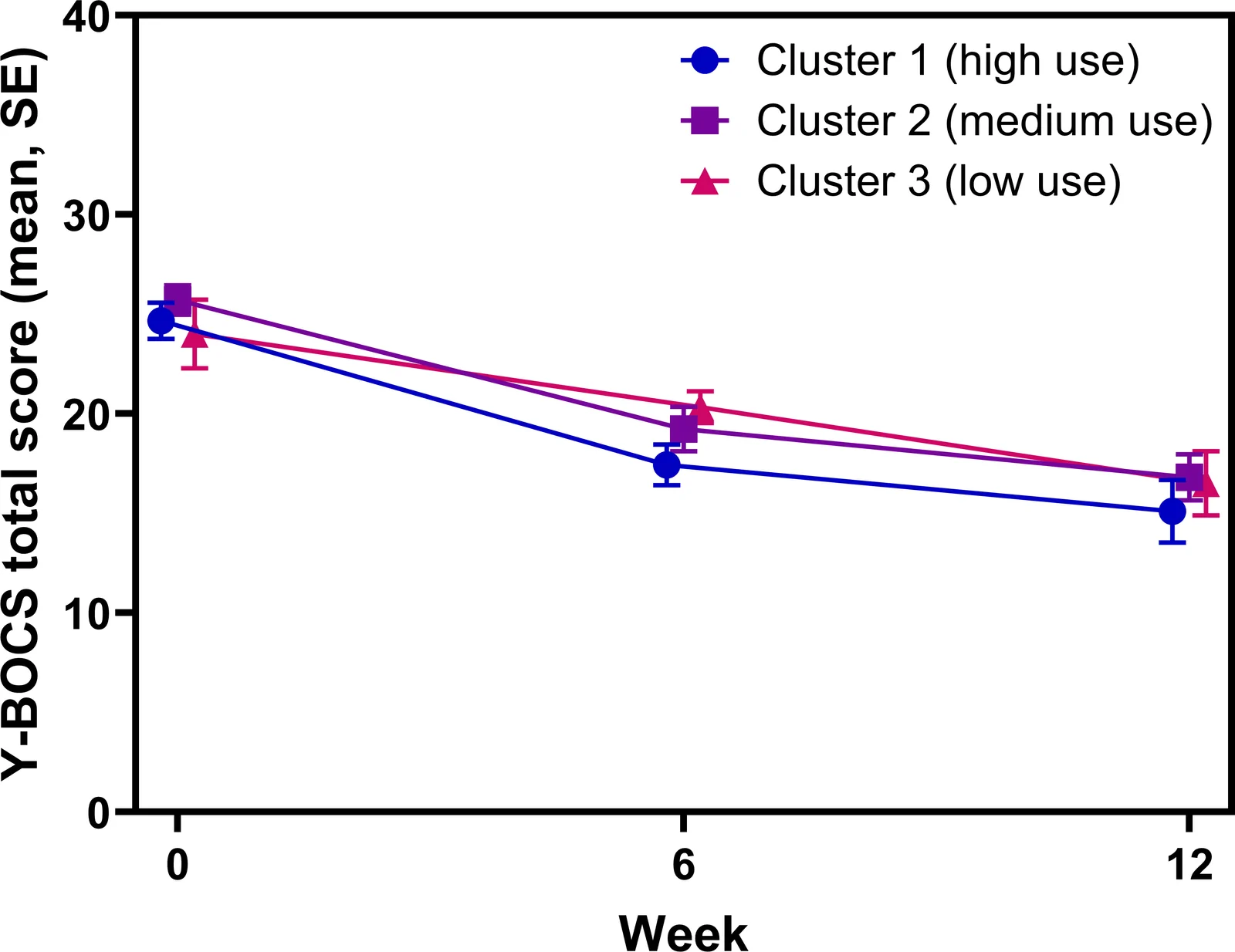
Severity of obsessive-compulsive disorder (OCD) symptoms (as measured by Y-BOCS total scores) over time during a 12-week smartphone cognitive behavioral therapy program for OCD in each of 3 identified app use clusters. Data points for clusters 1 and 3 are slightly offset to more clearly depict standard errors. Sample sizes are n=21 for cluster 1, n=31 for cluster 2, and n=8 for cluster 3. Y-BOCS: Yale-Brown Obsessive Compulsive Scale.

Descriptive differences in responder status emerged; cluster 1 had 43% (9/21) full responders, 24% (5/21) partial responders, and 33% (7/21) nonresponders; cluster 2 had 35% (11/31) full responders, 26% (8/31) partial responders, and 39% (12/31) nonresponders; and cluster 3 had 25% (2/8) full responders, 25% (2/8) partial responders, and 50% (4/8) nonresponders. Two of the 3 treatment dropouts, who were assumed to be nonresponders, were in cluster 3, while the third was in cluster 2. At posttreatment, clusters 1, 2, and 3 had 38% (8/21), 19% (6/31), and 13% (1/8) of participants in remission, respectively.

#### Aim 4: Examine Cluster Differences in Self-Reported Treatment Usage, Credibility and Expectancy, Satisfaction, and App Quality

##### Overview

Descriptive statistics and comparisons of participants’ self-reported treatment usage, credibility, expectancy, satisfaction, and quality are reported by cluster group in Table 2 (see Table S1 in Multimedia Appendix 8 for overall descriptives and Table S1 in Multimedia Appendix 6 for statistical detail).

##### Self-Reported Treatment Usage

For this outcome, we had limited interpretable data, particularly for users in cluster 3 (n=2 with interpretable self-report data). Thus, we focused on descriptive results for cluster 3 and only used tests of statistical significance for other clusters. Despite this, several notable patterns emerged. Compared with objective app use data, participants in cluster 1 tended to self-report much less app use (40% lower) than logs indicated. Participants in cluster 2 reported values close to those estimated from app logs (self-report 2% lower). Participants in cluster 3 also self-reported lower values than those estimated from app logs (self-report 38% lower). Participants in all clusters reported spending more time using skills off than on the app: cluster 1: 19% more, cluster 2: 99% more, and cluster 3: 63% more. The overall pattern of self-reported app use time across clusters was consistent with that calculated from app logs: cluster 1 reported the highest app use, cluster 2 an intermediate amount, and cluster 3 the least; the difference in self-reported app use time between clusters 1 and 2 was no longer significant after FDR adjustment (Table 2 and Table S1 in Multimedia Appendix 6).

##### Treatment Credibility and Expectancy

Participants’ baseline treatment credibility, measured after they were informed of their treatment condition, varied significantly across clusters. While we were unable to detect a significant difference in treatment credibility between clusters 1 and 2, cluster 3 perceived app CBT for OCD to be significantly less credible than cluster 2; this difference remained significant after FDR adjustment. Similar results were observed for treatment expectancy, although the difference between clusters 2 and 3 was no longer significant after FDR adjustment (Table 2).

##### Treatment Satisfaction and Perceived App Quality

These outcomes reflect subjective aspects of user engagement. Satisfaction results were similar to treatment credibility and expectancy, such that clusters 1 and 2 reported similar satisfaction at posttreatment, whereas cluster 3 reported significantly lower satisfaction than cluster 2 (and not different from cluster 1; the difference between clusters 2 and 3 was no longer significant after FDR adjustment; Table 2). Across clusters, participants perceived the app as very high in quality, with no significant differences across clusters in uMARS total scores or uMARS ratings of likelihood to recommend the app to others.

## Discussion

### Principal Findings

This study applied a previously developed approach for comprehensively measuring DMHI behavioral engagement [9] to a new clinical use case. This involved computing 21 objective behavioral engagement variables spanning use volume, frequency, breadth, depth, and temporal patterns. Three key findings emerged. First, overall engagement was exceptionally high and sustained over 12 weeks, contrasting with the rapid attrition commonly reported in DMHIs [7,35]. Second, cluster analysis revealed 3 distinct engagement profiles, multidimensionally high, moderate, and low engagers, yet all 3 groups experienced significant and similar reductions in OCD severity, challenging simple dose-response assumptions. Third, pre-treatment credibility was significantly associated with engagement profiles, even after FDR adjustment, identifying a potential modifiable target for improving DMHI engagement. These findings demonstrate that this measurement approach can be flexibly adapted across DMHIs and conditions, while also yielding novel insights specific to OCD.

### Comparison With Prior Work

Overall, participants’ total app use was high and exceeded the 77-175 minutes documented in prior DMHIs [36]. Use was also remarkably sustained over time; most users were still active at week 12. This pattern of minimal drop-off across 3 months contrasts sharply with usage documented in other DMHI studies; for instance, a report on PTSD Coach found that only 15.6% (26,110/166,861) of users reopened the app 1 week after downloading it [37]. These volume and retention results are particularly notable, given that OCD treatment centers on ERP, which is aversive and can trigger avoidance [13], especially in self-directed interventions that do not involve therapist support.

Several factors may explain this sustained engagement. First, the present data come from a clinical trial, whereas naturalistic studies capture in-the-wild downloads, representing 2 distinct contexts [38]. Clinical trial participants may be more motivated through self-selection and less psychiatrically complex due to eligibility criteria. They may benefit from reinforcing factors such as study staff contact for assessments. That said, the current pattern of sustained use stands out even relative to our prior randomized controlled trial of smartphone CBT for BDD, which showed active user rates declining from 97% to 27% between weeks 1 and 12 [9]. Thus, sustained engagement in this sample likely extends beyond the effects of clinical trials alone. Another potential factor is the intervention itself. In smartphone CBT for BDD, exposure focuses narrowly on facing situations that trigger appearance concerns. After several weeks of practice, the content of BDD exposures may inherently lack novelty, and users may have less need to interact with the app. On the other hand, CBT for OCD can include widely varying exposures across symptom domains, depending on the user’s clinical presentation. Thus, participants may have returned to Perspectives OCD over time because they continued to benefit from novel content. Finally, it is possible that personality traits associated with OCD, such as conscientiousness [39,40], responsibility [41], perfectionism [42], or need for completion [43], may translate to diligence in completing in-app assignments, although the literature on this is mixed.

The observed pattern of brief and frequent app use suggests that users engage with DMHIs through short interactions rather than the 45‐ to 50-minute concentrated doses typical of traditional psychotherapy [44,45]. This pattern was consistent with our previous findings for BDD [9]. This distinct DMHI usage pattern suggests that app content should be designed to deliver meaningful information and skills practice in brief increments, reinforced through frequent use over time.

Cluster analysis identified 3 user profiles that differed across all measured dimensions. Cluster 1 users showed extensive engagement across all dimensions, including ERP. Cluster 2 users demonstrated moderate, consistent use, completing core content (median 7 ERP exercises) without exhaustive exploration. Cluster 3, despite spending meaningful time on the app, completed few to no ERP exercises and showed a sharp temporal decline in use over time. In contrast to hypotheses, all clusters experienced significant reductions in OCD severity, and there were no statistically significant differences in the magnitude of symptom change by cluster. On the other hand, while we did not analyze response and remission rate differences statistically, rates of full response were descriptively higher in cluster 1 than in cluster 2 or 3, and rates of remission appeared to be considerably higher in cluster 1 than in cluster 2. This pattern of findings is similar to our prior BDD study, which showed a nonsignificant trend toward deeper use being associated with greater improvement [9]. Nonsignificant results may in part reflect the small sample size, particularly in cluster 3, which limited our power to detect what may be clinically relevant differences. Indeed, at a prevalence rate of 40% and a 3:1 group size ratio (ie, assuming participants with very low app use make up only one-quarter of the sample), a sample size of n=224 would be needed to detect a 20% difference in response rates at 80% power. Nonsignificant results also align with broader literature that has inconsistently documented dose-response relationships between engagement and DMHI outcomes [46-48]. These findings invite careful consideration of what constitutes “effective” engagement. One possible interpretation of results is that, when an intervention is potent, as this one appears to be [16], multiple styles of use may be effective. For instance, while ERP is the frontline strategy for OCD, evidence also supports the benefits of other skills such as cognitive strategies for OCD [49,50]. Although cluster 3 users completed few, if any, exposures, they may have benefited from other app content. To this end, 1 potential benefit to self-directed DMHIs is that users who are not willing to engage in certain treatment strategies (eg, ERP) can choose to learn other skills, which may also be therapeutic; in other words, self-directed digital interventions allow users to gravitate toward content that aligns with their readiness.

That said, these null findings may also reflect that engagement was relatively high even within cluster 3, whose median use exceeded that reported in unguided internet-based OCD treatments (129 minutes [46]). A small sample alongside a restricted range of engagement and outcomes may have prevented us from observing what happens to truly disengaged users. This restricted range is in part due to data coming from an efficacy trial; if replicated in the wild, we expect to find greater variability in engagement and treatment outcomes, giving more range from which to identify effective and ineffective engagement styles. Testing the current measurement approach in large real-world data from implemented interventions is a critical next step in the current program of research.

To supplement objective measures of engagement, we asked participants to self-report the time they spent practicing skills both within and outside the app, a complementary variable referred to as *enactment* in prior DMHI engagement research [18]. These data had limitations related to missingness and data integrity. Despite this, they offered preliminary insights that would be impossible to infer from app analytics alone. All clusters reported spending more time using skills off the app than on the app. This finding underscores the value of asking such a question, as ultimately, the goal is for users to develop skills that they can use independently in daily life. Although we had data from only 2 users in cluster 3, their responses to this item shed further light on why these participants may have improved significantly despite having interacted with the app the least. Cluster 3 participants, in contrast to those in cluster 1, may include people who preferred to visit materials briefly on the app and then implement skills off the app in daily life, still reaping therapeutic benefits despite reduced observed behavioral engagement.

A novel contribution of this study was its incorporation of participants’ baseline treatment credibility and expectancy scores. While clusters 1 and 2 perceived app CBT for OCD to be similarly credible and had comparable treatment expectations, cluster 3 perceived treatment to be significantly less credible and had lower expectations than cluster 2; the difference in credibility (but not expectancy) ratings remained significant after FDR adjustment. Results are consistent with research showing significant associations between treatment expectancy and adherence [51,52]. Relatedly, recent research on OCD has shown that treatment credibility and expectancy predict outcomes in concentrated exposure-based treatment [14]. It is possible that those who most strongly believe in the treatment’s premise and who have high expectations that treatment will work are most likely to engage deeply and in a targeted way (eg, focused on ERP modules), and this deeper engagement may be a mechanism through which credibility and expectancy predict outcomes. Current results are correlational; we cannot conclude that higher credibility or expectancy *caused* greater engagement. If further experimental studies confirm a causal relationship, these novel results highlight potential intervention targets to enhance engagement and, potentially, outcomes [53]. App developers can boost credibility by providing a detailed rationale for the treatment approach during onboarding and partnering with credible codevelopers, such as professional experts. To enhance expectancy, psychoeducational content can instill hope by providing information about anticipated outcomes (drawing on the literature).

End-of-treatment satisfaction also varied by cluster, with cluster 3, the least engaged participants, reporting significantly lower treatment satisfaction than cluster 2. Interestingly, all clusters gave the app similar, high-quality ratings on the uMARS, suggesting that lower satisfaction in cluster 3 reflected aspects of the treatment experience beyond app quality. Results linking lower satisfaction ratings to reduced behavioral engagement begin to address a gap identified in recent reviews [3,6], which have noted that prior DMHI studies tend to examine either subjective or behavioral engagement, in isolation.

### Strengths and Limitations

This study has several strengths, including the demonstration that a comprehensive behavioral engagement measurement approach generalizes across clinical samples, the integration of objective and select subjective engagement measures to provide a more complete picture than either alone, and the novel examination of pretreatment credibility and expectancy as predictors of objective engagement patterns. Additionally, several limitations should be noted. This was an exploratory, non-preregistered secondary analysis of an efficacy trial, which included eligibility criteria and participant interactions with research staff, thereby limiting its generalizability to real-world implementation contexts. High engagement and strong treatment outcomes across clusters likely in part reflect this and create a restriction of range that may have obscured engagement-outcome associations. The sample was relatively small, affecting the stability of the cluster solution and homogeneity of the chosen clusters. Although a bootstrap stability analysis of our sample indicated reasonable cluster stability, analyses of larger samples of app users are needed. Relatedly, the small size of cluster 3 (n=8), in particular, limits statistical power for comparisons involving this group. The number of tests of statistical significance may have resulted in type I errors. We sought to mitigate this risk by also evaluating *P* values adjusted for multiple testing via an FDR adjustment. Although comprehensive in its treatment of behavioral engagement, our approach does not capture subjective dimensions such as cognitive absorption or emotions during use [4]. We incorporated posttreatment satisfaction and quality ratings, which reflect user attitudes about the DMHI, but future studies should additionally measure subjective cognitive and affective engagement during use (eg, via ecological momentary assessment, in-the-moment ratings of willingness, and affective participation). This gap may be especially important for a DMHI targeting OCD; as noted previously, ERP is aversive, and one’s willingness to actively participate and experience anxiety during ERP is critical. It is possible that the 3 clusters differed in their attentional, cognitive, and affective participation (and conversely, experiential avoidance) during app-delivered ERP, and capturing these variables might further clarify results related to treatment outcomes across clusters. Additionally, OCD is a heterogeneous condition, and this study was not powered to examine whether symptom subtypes or the number of symptom domains endorsed influenced engagement profiles. Replication in large, real-world samples is a critical next step.

### Conclusions

This work demonstrates that our approach to comprehensive behavioral engagement measurement is feasible and informative across different DMHIs. The methodological approach provides a nuanced view of the quantity and quality of users’ behavioral engagement with digital tools that researchers can adapt to their own DMHIs. The novel findings that pretreatment credibility and expectancy are associated with different behavioral engagement patterns identify an actionable target to potentially boost behavioral engagement. Based on our experience across 2 DMHIs, we recommend that researchers measuring engagement include, at minimum, variables capturing use volume (eg, total time and number of sessions), temporal patterns (eg, consistency of use over treatment phases and inactivity periods), feature-level engagement (eg, completion of therapeutic content theorized to be most active for the given clinical target), and session characteristics (eg, duration distribution). We further recommend supplementing objective metrics with self-reported off-app skills practice, pre- and posttreatment attitudes, and measures of cognitive and affective engagement, alongside behavioral engagement.

## Supplementary material

10.2196/98129 Multimedia Appendix 1 STROBE (Strengthening the Reporting of Observational Studies in Epidemiology) statement—checklist of items that should be included in reports of observational studies (von Elm et al, 2007).

10.2196/98129 Multimedia Appendix 2 Heatmap of Pearson correlations between all app use variables used in the clustering process. Continuous (including integer count) variables (eg, minutes of app use time, number of coach messages sent, etc) were rescaled from 0 to 1. Intensifying green colors indicate positive correlations, intensifying red colors indicate negative correlations, and white or near white colors are correlations near zero. Variables are arranged in the same order (top to bottom) as presented in Table 1 (main manuscript).

10.2196/98129 Multimedia Appendix 3 Inverse scree plot based on semipartial *R*^2^ in the hierarchical cluster solution using Ward’s method with Euclidean distances. For ease of interpretation and maintaining reasonable cluster sizes, we chose a cluster number of 3.

10.2196/98129 Multimedia Appendix 4 Cluster result dendrogram with *P* values based on multiscale bootstrap resampling (R package pvclust; Suzuki et al, 2019; Suzuki and Shimodaira, 2006). *P* values printed in red color are the approximately unbiased *P* values, while *P* values printed in green color are “bootstrap probability” values. Clusters with high approximately unbiased values (eg, 95%, marked by thin red boxes) are strongly supported by data. As presented in the results, cluster 1 is delineated with a thick blue line box, cluster 2 with a thick purple box, and cluster 3 with a thick red box.

10.2196/98129 Multimedia Appendix 5 Statistical details for the analysis of app use cluster differences reported in Table 1.

10.2196/98129 Multimedia Appendix 6 Statistical details for the analysis of cluster differences in participant-reported treatment engagement, credibility, expectancy, and satisfaction reported in Table 2.

10.2196/98129 Multimedia Appendix 7 Overall (whole sample) app use characteristics of participants (n=60) in a trial studying the efficacy of a 12-week smartphone cognitive behavioral therapy program for obsessive compulsive disorder.

10.2196/98129 Multimedia Appendix 8 Overall (whole sample, n=60) participant-reported treatment engagement, credibility, expectancy, and satisfaction reported in Table 2.

## Abbreviations

BDD: body dysmorphic disorder
CBT: cognitive behavioral therapy
CGI-I: Clinical Global Impressions–Improvement scale
CSQ-8: 8-item Client Satisfaction Questionnaire
DMHI: digital mental health intervention
*DSM-5*: *Diagnostic and Statistical Manual* of *Mental Disorders* (Fifth Edition)
ERP: exposure with response prevention
ES: effect size
FDR: false discovery rate
IE: independent evaluator
LSM: least-squares mean
MINI: Mini International Neuropsychiatric Interview
OCD: obsessive compulsive disorder
STROBE: Strengthening the Reporting of Observational Studies in Epidemiology
uMARS: user version of the Mobile Application Rating Scale
Y-BOCS: Yale-Brown Obsessive Compulsive Scale
