# Supplementary material for: Characterizing Behavioral Engagement in Smartphone Cognitive Behavioral Therapy for Obsessive Compulsive Disorder: Secondary Analysis of a Randomized Controlled Trial
Source: JMIR Form Res. 2026 Oct 19;10:e98129. doi: 10.2196/98129 (PMC13679862; doi:10.2196/98129)
Supplement: Supplementary file 1 [file formative-v10-e98129-s001.docx]

**Multimedia Appendix 1**

**STROBE Statement—checklist of items that should be included in reports of observational studies (Von Elm et al., 2007)**

|  | **Item No.** | **Recommendation** | **Page No.** | **Relevant text from manuscript** |
| --- | --- | --- | --- | --- |
| **Title and abstract** | 1 | (*a*) Indicate the study’s design with a commonly used term in the title or the abstract | Title; Abstract (Methods) | Abstract Methods: “This exploratory secondary analysis… cluster analysis…”; title states “A Cluster Analysis of 21 Objective App Use Metrics.” NOTE: consider adding “secondary analysis” / “observational” term to title. |
|  |  | (*b*) Provide in the abstract an informative and balanced summary of what was done and what was found | Abstract | Structured abstract (Background, Objective, Methods, Results, Conclusions) reports design, N=60, the four aims, the three-cluster solution, and outcomes. |
| **Introduction** | | | | |
| **Background/rationale** | 2 | Explain the scientific background and rationale for the investigation being reported | Introduction (paras 1–5) | Introduction: engagement is multidimensional and under-measured; prior BDD roadmap [9]; rationale for extending to OCD (provider shortage, ERP aversiveness). |
| **Objectives** | 3 | State specific objectives, including any prespecified hypotheses | Introduction (final para) | Four numbered aims stated; hypotheses that greater/ERP engagement would relate to greater improvement and more favorable ratings, expected to be modest. |
| **Methods** | | | | |
| **Study design** | 4 | Present key elements of study design early in the paper | Methods – Overview and study design | “This is a secondary analysis of a 12-week randomized controlled trial… only data from the Perspectives OCD condition were used (N=60).” |
| **Setting** | 5 | Describe the setting, locations, and relevant dates, including periods of recruitment, exposure, follow-up, and data collection | Methods – Participants; Procedures | Recruited nationally from the MGH Center for OCD and Related Disorders (social media, professional organizations, referrals), July 2021–July 2023; assessments at baseline, week 6, week 12. |
| **Participants** | 6 | (*a*) *Cohort study*—Give the eligibility criteria, and the sources and methods of selection of participants. Describe methods of follow-up | Methods – Participants; Procedures | Eligibility criteria listed (adults ≥18, US, primary OCD via MINI, owned smartphone; exclusions). Followed over 12 weeks with assessments at baseline/week 6/week 12. |
|  |  | (*b*) *Cohort study*—For matched studies, give matching criteria and number of exposed and unexposed | N/A | Not applicable – single-group secondary analysis (no matching). |
| **Variables** | 7 | Clearly define all outcomes, exposures, predictors, potential confounders, and effect modifiers. Give diagnostic criteria, if applicable | Methods – Measures; App engagement | 21 objective engagement variables defined across five categories; outcome = Y-BOCS; subjective predictors (CEQ credibility/expectancy, CSQ-8, uMARS). OCD diagnosis via MINI (DSM-5). |
| **Data sources/ measurement** | 8 | For each variable of interest, give sources of data and details of methods of assessment (measurement). Describe comparability of assessment methods if there is more than one group | Methods – Measures; App engagement; Self-report | App analytics passively logged by the app; clinician-rated Y-BOCS/CGI-I by blinded IEs; self-reports via REDCap. Internal consistencies reported for scales. |
| **Bias** | 9 | Describe any efforts to address potential sources of bias | Methods – Procedures; Analytic Approach | Blinded independent evaluators; concealed allocation in parent trial; outlier capping rules for app actions; FDR adjustment for multiple comparisons. |
| **Study size** | 10 | Explain how the study size was arrived at | Methods – Overview; Discussion | N=60 = all Perspectives OCD participants in the parent RCT (secondary analysis; no separate sample-size calculation). Power discussed in Discussion (e.g., n=224 needed to detect a 20% response difference). |
| **Quantitative variables** | 11 | Explain how quantitative variables were handled in the analyses. If applicable, describe which groupings were chosen and why | Methods – App engagement; Analytic Approach (Aim 2) | Variables rescaled 0–1 (min-max / proportions) for clustering; session duration binned into four categories; outlier handling described; Y-BOCS severity categories defined. |
| **Statistical methods** | 12 | (*a*) Describe all statistical methods, including those used to control for confounding | Methods – Analytic Approach (Aims 1–4) | Hierarchical agglomerative clustering (Ward’s, squared Euclidean); ANOVA / Wilcoxon for cluster differences; hierarchical generalized linear mixed model for Y-BOCS over time with stratification covariate. |
|  |  | (*b*) Describe any methods used to examine subgroups and interactions | Methods – Analytic Approach (Aim 3) | Time-by-cluster and stratification-by-time interactions modeled as fixed effects; specific contrasts for between-cluster change. |
|  |  | (*c*) Explain how missing data were addressed | Methods – Analytic Approach; Measures | Mixed model used all available data via maximum likelihood; dropouts (3/60) coded non-responders; CSQ-8/uMARS missing for 7/60 excluded for those outcomes; missingness on self-report utilization quantified. |
|  |  | (*d*) *Cohort study*—If applicable, explain how loss to follow-up was addressed | Methods – Analytic Approach | Loss to follow-up handled via maximum likelihood estimation on all available data; three dropouts coded as non-responders for response/remission. |
|  |  | (*e*) Describe any sensitivity analyses | Methods – Analytic Approach; Supplement | FDR-adjusted P-values reported alongside raw values (Tables 1–2, Supplementary Tables S1–S2); cluster stability assessed via bootstrap (Supplementary Figure S3). |
| **Results** | | | | |
| **Participants** | 13 | (a) Report numbers of individuals at each stage of study—eg numbers potentially eligible, examined for eligibility, confirmed eligible, included, completing follow-up, and analysed | Results – Sample Description; Aim 3 | All 60 Perspectives OCD participants had app data and were analyzed; 3/60 dropped out; active users tracked weekly (60/60 week 1 to 52/60 week 12). Full CONSORT flow in parent trial [14]. |
|  |  | (b) Give reasons for non-participation at each stage | Methods – Measures; Results | Reasons for missing self-report data given (dropout, skipped/missed assessments, non-reconcilable percentages). |
|  |  | (c) Consider use of a flow diagram | Parent trial [14] | CONSORT flow diagram presented in the primary trial paper [14]; not duplicated here per JMIR (avoid redundancy). |
| **Descriptive data** | 14 | (a) Give characteristics of study participants and information on exposures and potential confounders | Results – Sample Description; Table 1 | Age M(SD) 39.0 (14.7); gender, race/ethnicity, baseline Y-BOCS distribution reported; full app-use descriptives in Table 1. |
|  |  | (b) Indicate number of participants with missing data for each variable of interest | Methods – Measures; Results | Missingness quantified: CSQ-8/uMARS 7/60; self-report utilization ~32%; dropouts 3/60. |
|  |  | (c) *Cohort study*—Summarise follow-up time | Methods; Results | Fixed 12-week follow-up window for all participants; assessments at baseline, week 6, week 12. |
| **Outcome data** | 15 | *Cohort study*—Report numbers of outcome events or summary measures over time | Results – Aim 3; Figure 2 | Y-BOCS least-square means over time by cluster reported with 95% CIs; response/remission counts per cluster given descriptively. |
| **Main results** | 16 | (*a*) Give unadjusted and, if applicable, confounder-adjusted estimates and their precision. Make clear which confounders were adjusted for | Results – Aims 1–4; Tables 1–2 | Cluster comparisons reported with effect sizes (Hedges g) and 95% CIs; mixed model adjusted for stratification (baseline medication status); raw and FDR-adjusted P-values. |
|  |  | (*b*) Report category boundaries when continuous variables were categorized | Methods – Measures; App engagement | Y-BOCS severity bands (mild 0–13, moderate 14–25, moderate-severe 26–34, severe 35–40) and session-duration bins (10s–3min, >3–5, >5–10, >10) defined. |
|  |  | (*c*) If relevant, consider translating estimates of relative risk into absolute risk | N/A | Not applicable – no relative-risk estimates reported. |
| **Other analyses** | 17 | Report other analyses done—eg analyses of subgroups and interactions, and sensitivity analyses | Results – Aims 2–4; Supplement | Cluster-difference tests, FDR-adjusted analyses, and bootstrap cluster-stability analysis (Supplementary Materials). |
| **Discussion** | | | | |
| **Key results** | 18 | Summarise key results with reference to study objectives | Discussion – Principal findings | Three findings summarized: high/sustained engagement; three profiles with similar symptom reduction; pre-treatment credibility associated with engagement. |
| **Limitations** | 19 | Discuss limitations of the study, taking into account sources of potential bias or imprecision. Discuss both direction and magnitude of any potential bias | Discussion – Strengths and limitations | Exploratory non-preregistered secondary analysis; range restriction from efficacy trial; small sample / small Cluster 3 (n=8); multiple tests (Type I risk, mitigated by FDR); behavioral-only engagement. |
| **Interpretation** | 20 | Give a cautious overall interpretation considering objectives, limitations, multiplicity of analyses, results from similar studies, and other relevant evidence | Discussion – Comparison with prior work; Conclusions | Cautious interpretation that multiple engagement paths may be beneficial; situated against BDD roadmap [9] and DMHI dose-response literature; notes correlational (non-causal) nature of credibility findings. |
| **Generalisability** | 21 | Discuss the generalisability (external validity) of the study results | Discussion – Comparison with prior work; Limitations | Notes efficacy-trial context limits generalizability to real-world implementation; calls for replication in large, real-world samples. |
| **Other information** | | | | |
| **Funding** | 22 | Give the source of funding and the role of the funders for the present study and, if applicable, for the original study on which the present article is based | Funding; Conflicts of Interest | Funded by Lee Baer Fund, Hope Fund, and an anonymous donor (Wilhelm, PI). Parent trial funded by Koa Health, whose role/COI is described in the Conflicts of Interest section. |

Von Elm, E., Altman, D. G., Egger, M., Pocock, S. J., Gøtzsche, P. C., & Vandenbroucke, J. P. (2007). The Strengthening the Reporting of Observational Studies in Epidemiology (STROBE) statement: guidelines for reporting observational studies. The lancet, 370(9596), 1453-1457.
