# Supplementary material for: Characterizing Behavioral Engagement in Smartphone Cognitive Behavioral Therapy for Obsessive Compulsive Disorder: Secondary Analysis of a Randomized Controlled Trial
Source: JMIR Form Res. 2026 Oct 19;10:e98129. doi: 10.2196/98129 (PMC13679862; doi:10.2196/98129)
Supplement: Supplementary file 2 [file formative-v10-e98129-s002.docx]

**Multimedia Appendix 2**

**Figure S1**. Heatmap of Pearson correlations between all app use variables used in the clustering process. Continuous (including integer count) variables (e.g., minutes of app use time, number of coach messages sent, etc.) were rescaled from 0 to 1. Intensifying green colors indicate positive correlations, intensifying red colors indicate negative correlations, and white or near white colors are correlations near zero. Variables are arranged in the same order (top to bottom) as presented in Table 1 (main manuscript).
