# Supplementary material for: Characterizing Behavioral Engagement in Smartphone Cognitive Behavioral Therapy for Obsessive Compulsive Disorder: Secondary Analysis of a Randomized Controlled Trial
Source: JMIR Form Res. 2026 Oct 19;10:e98129. doi: 10.2196/98129 (PMC13679862; doi:10.2196/98129)
Supplement: Supplementary file 3 [file formative-v10-e98129-s003.docx]

**Multimedia Appendix 3**

**Figure S1**. Inverse scree plot based on semi-partial r-squared in the hierarchical cluster solution using Ward’s method with Euclidean distances. For ease of interpretation and maintaining reasonable cluster sizes, we chose a cluster number of 3.
