# Supplementary material for: Characterizing Behavioral Engagement in Smartphone Cognitive Behavioral Therapy for Obsessive Compulsive Disorder: Secondary Analysis of a Randomized Controlled Trial
Source: JMIR Form Res. 2026 Oct 19;10:e98129. doi: 10.2196/98129 (PMC13679862; doi:10.2196/98129)
Supplement: Supplementary file 4 [file formative-v10-e98129-s004.docx]

**Multimedia Appendix 4**

**Figure S1**. Cluster result dendrogram with *P*-values based on multiscale bootstrap resampling (R package pvclust; Suzuki et al., 2019; Suzuki and Shimodaira, 2006). AU *P*-values (printed in red color) are the approximately unbiased p-values, while the BP *P*-values (printed in green color) are "bootstrap probability" values. Clusters with high AU values (e.g., 95%, marked by thin red boxes) are strongly supported by data. As presented in the results, Cluster 1 is delineated with a thick blue line box, Cluster 2 with a thick purple box, and Cluster 3 with a thick red box.

Suzuki R, Terada Y, Shimodaira H (2019). pvclust: Hierarchical Clustering with P-Values via Multiscale Bootstrap Resampling. R package version 2.2-0, https://CRAN.R-project.org/package=pvclust.

Suzuki, R. and Shimodaira, H. (2006) "Pvclust: an R package for assessing the uncertainty in hierarchical clustering", Bioinformatics, 22 (12): 1540-1542.
