# Supplementary material for: Characterizing Behavioral Engagement in Smartphone Cognitive Behavioral Therapy for Obsessive Compulsive Disorder: Secondary Analysis of a Randomized Controlled Trial
Source: JMIR Form Res. 2026 Oct 19;10:e98129. doi: 10.2196/98129 (PMC13679862; doi:10.2196/98129)
Supplement: Supplementary file 5 [file formative-v10-e98129-s005.docx]

**Multimedia Appendix 5**

**Table S1**. Statistical details for the analysis of app use cluster differences reported in Table 1.

| **App use variables** | | | **Test** | ***Z* statistic (*n_1_, n_2_*)** | **LSMeans Diff. [95% CI]** | | | ***t* test (*df*)** | **raw *P*** | **FDR *P*** |
| --- | --- | --- | --- | --- | --- | --- | --- | --- | --- | --- |
| ***Comparison of Cluster 1 vs. Cluster 2*** | | |  |  |  |  |  |  |  |  |
|  | Amount (volume) of usage | | |  |  |  |  |  |  |  |
|  |  | Total app use, minutes | Wilcoxon | 5.73 (21, 31) |  |  |  |  | <.001 | <.001 |
|  |  | Total number of app sessions | ANOVA |  | 47.0 | [33.7, | 60.4] | 7.05 (57) | <.001 | <.001 |
|  |  | Total number of days of app use | ANOVA |  | 19.8 | [12.5, | 27.2] | 5.40 (57) | <.001 | <.001 |
|  | Frequency and inactivity periods | | |  |  |  |  |  |  |  |
|  |  | App use frequency (inactivity), log-mins | ANOVA |  | -0.7 | [-0.9, | -0.4] | -5.32 (57) | <.001 | <.001 |
|  |  | Number of inactivity periods of 7+ days | Wilcoxon | -3.9 (21, 31) |  |  |  |  | <.001 | <.001 |
|  | Use of key app features | |  |  |  |  |  |  |  |  |
|  |  | % of main modules completed | Wilcoxon | 4.18 (21, 31) |  |  |  |  | <.001 | <.001 |
|  |  | % of main exercises completed | Wilcoxon | 4.23 (21, 31) |  |  |  |  | <.001 | <.001 |
|  |  | % of all exercises (started) completed | Wilcoxon | 1.56 (21, 31) |  |  |  |  | .12 | .16 |
|  |  | % of ERP exercises (started) completed | Wilcoxon | 1.61 (21, 31) |  |  |  |  | .11 | .15 |
|  |  | Number of ERP exercises completed | Wilcoxon | 5.79 (21, 31) |  |  |  |  | <.001 | <.001 |
|  |  | Number of custom ERP exercises created | Wilcoxon | 1.26 (21, 31) |  |  |  |  | .21 | .25 |
|  |  | Number of messages sent to the coach | ANOVA |  | 9.2 | [4.5, | 14.0] | 3.91 (57) | <.001 | .001 |
|  | Duration of sessions | |  |  |  |  |  |  |  |  |
|  |  | % of sessions >10 sec. to 3 min. | ANOVA |  | -11.2 | [-19.0, | -3.4] | -2.88 (57) | .01 | .01 |
|  |  | % of sessions >3 min. to 5 min. | ANOVA |  | -1.3 | [-4.1, | 1.5] | -0.92 (57) | .36 | .41 |
|  |  | % of sessions >5 min. to 10 min. | ANOVA |  | 0.1 | [-3.2, | 3.5] | 0.07 (57) | .95 | .95 |
|  |  | % of sessions >10 min. | ANOVA |  | 12.4 | [5.2, | 19.6] | 3.45 (57) | .001 | .002 |
|  |  | Entropy of session duration | Wilcoxon | 1.42 (21, 31) |  |  |  |  | .16 | .19 |
|  |  | Median session duration | Wilcoxon | 2.46 (21, 31) |  |  |  |  | .01 | .02 |
|  | Consistency of use over time | |  |  |  |  |  |  |  |  |
|  |  | % of app min. in weeks 1-4 | ANOVA |  | -11.8 | [-22.4, | -1.2] | -2.24 (57) | .03 | .05 |
|  |  | % of app min. in weeks 5-8 | ANOVA |  | 1.8 | [-6.0, | 9.5] | 1.97 (57) | .65 | .68 |
|  |  | % of app min. in weeks 9-12 | ANOVA |  | 10.03 | [3.0, | 17.1] | 2.86 (57) | .01 | .01 |

**Table S1 (continued)**. Statistical details for the analysis of app use cluster differences reported in Table 1.

| **App use variables** | | | **Test** | ***Z* statistic (*n_1_, n_2_*)** | **LSMeans Diff [95% CI]** | ***t* test (*df*)** | **raw *P*** | **FDR *P*** |
| --- | --- | --- | --- | --- | --- | --- | --- | --- |
| ***Comparison of Cluster 2 vs. Cluster 3*** | | | |  |  |  |  |  |
|  | Amount (volume) of usage | |  |  |  |  |  |  |
|  |  | Total app use, minutes | Wilcoxon exact | -2.24 (31, 8) |  |  | .02 | .04 |
|  |  | Total number of app sessions | ANOVA |  | 11.8 [-6.9, 30.6] | 1.26 (57) | .21 | .25 |
|  |  | Total number of days of app use | ANOVA |  | 9.2 [-1.1, 19.5] | 1.79 (57) | .08 | .12 |
|  | Frequency and inactivity periods | |  |  |  |  |  |  |
|  |  | App use frequency (inactivity), log-mins | ANOVA |  | -0.5 [-0.8,-0.1] | -2.58 (57) | .01 | .02 |
|  |  | Number of inactivity periods of 7+ days | Wilcoxon exact | 0.48 (31, 8) |  |  | .64 | .68 |
|  | Use of key app features | |  |  |  |  |  |  |
|  |  | % of main modules completed | Wilcoxon exact | -2.64 (31, 8) |  |  | .007 | .01 |
|  |  | % of main exercises completed | Wilcoxon exact | -3.85 (31, 8) |  |  | <.001 | .000 |
|  |  | % of all exercises (started) completed | Wilcoxon exact | -2.62 (31, 8) |  |  | .007 | .01 |
|  |  | % of ERP exercises (started) completed | Wilcoxon exact | -4.20 (31, 8) |  |  | <.001 | .000 |
|  |  | Number of ERP exercises completed | Wilcoxon exact | -3.96 (31, 8) |  |  | <.001 | .000 |
|  |  | Number of custom ERP exercises created | Wilcoxon | -1.17 (31, 8) |  |  | .43 | .47 |
|  |  | Number of messages sent to the coach | ANOVA |  | 3.9 [-2.7, 10.6] | 1.18 (57) | .24 | .28 |
|  | Duration of sessions | |  |  |  |  |  |  |
|  |  | % of sessions >10 sec. to 3 min. | ANOVA |  | -9.2 [-20.1, 1.7] | -1.69 (57) | .10 | .14 |
|  |  | % of sessions >3 min. to 5 min. | ANOVA |  | 2.9 [-1.0, 6.9) | 1.48 (57) | .15 | .19 |
|  |  | % of sessions >5 min. to 10 min. | ANOVA |  | -0.6 [-5.3, 4.1] | -0.24 (57) | .81 | .84 |
|  |  | % of sessions >10 min. | ANOVA |  | 6.8 [-3.2, 16.9] | 1.36 (57) | .18 | .22 |
|  |  | Entropy of session duration | Wilcoxon exact | -2.10 (31, 8) |  |  | .03 | .05 |
|  |  | Median session duration | Wilcoxon exact | -1.83 (31, 8) |  |  | .07 | .10 |
|  | Consistency of use over time | |  |  |  |  |  |  |
|  |  | % of app minutes in weeks 1-4 | ANOVA |  | -17.1 [-32.0, -2.3] | -2.31 (57) | .02 | .04 |
|  |  | % of app minutes in weeks 5-8 | ANOVA |  | 9.5 [-1.4, 20.3] | 1.74 (57) | .09 | .13 |
|  |  | % of app minutes in weeks 9-12 | ANOVA |  | 7.68 [-2.2, 17.5] | 1.56 (57) | .12 | .16 |

**Table S1 (continued)**. Statistical details for the analysis of app use cluster differences reported in Table 1.

| **App use variables** | | | **Test** | ***Z* statistic (*n_1_, n_2_*)** | **LSMeans Diff [95% CI]** | ***t* test (*df*)** | **raw *P*** | **FDR *P*** |
| --- | --- | --- | --- | --- | --- | --- | --- | --- |
| ***Comparison of Cluster 1 vs. Cluster 3*** | | | |  |  |  |  |  |
|  | Amount (volume) of usage | |  |  |  |  |  |  |
|  |  | Total app use, minutes | Wilcoxon exact | -4.07 (21, 8) |  |  | <.001 | .000 |
|  |  | Total number of app sessions | ANOVA |  | 58.9 [39.2, 78.5] | 6.00 (57) | <.001 | .000 |
|  |  | Total number of days of app use | ANOVA |  | 29.0 [18.2, 39.8] | 5.37 (57) | <.001 | .000 |
|  | Frequency and inactivity periods | |  |  |  |  |  |  |
|  |  | App use frequency (inactivity), log-mins | ANOVA |  | -1.1 [-1.5, -0.8] | -6.08 (57) | <.001 | .000 |
|  |  | Number of inactivity periods of 7+ days | Wilcoxon exact | 3.43 (21, 8) |  |  | .000 | .001 |
|  | Use of key app features | |  |  |  |  |  |  |
|  |  | % of main modules completed | Wilcoxon exact | -4.09 (21, 8) |  |  | <.001 | .000 |
|  |  | % of main exercises completed | Wilcoxon exact | -4.14 (21, 8) |  |  | <.001 | .000 |
|  |  | % of all exercises (started) completed | Wilcoxon exact | -2.89 (21, 8) |  |  | .002 | .005 |
|  |  | % of ERP exercises (started) completed | Wilcoxon exact | -4.17 (21, 8) |  |  | <.001 | .000 |
|  |  | Number of ERP exercises completed | Wilcoxon exact | -4.09 (21, 8) |  |  | <.001 | .000 |
|  |  | Number of custom ERP exercises created | Wilcoxon exact | -1.62 (21, 8) |  |  | .15 | .19 |
|  |  | Number of messages sent to the coach | ANOVA |  | 13.2 [6.2, 20.1] | 3.79 (57) | .000 | .001 |
|  | Duration of sessions | |  |  |  |  |  |  |
|  |  | % of sessions >10 sec. to 3 min. | ANOVA |  | -20.4 [-31.8, -8.9] | -3.57 (57) | .001 | .002 |
|  |  | % of sessions >3 min. to 5 min. | ANOVA |  | 1.6 [-2.5, 5.8] | 0.78(57) | .44 | .47 |
|  |  | % of sessions >5 min. to 10 min. | ANOVA |  | -0.5 [-5.4, 4.5] | -0.18 (57) | .85 | .87 |
|  |  | % of sessions >10 min. | ANOVA |  | 19.2 [8.7, 29.8[ | 3.64 (57) | .001 | .002 |
|  |  | Entropy of session duration | Wilcoxon exact | -3.20 (21, 8) |  |  | .001 | .002 |
|  |  | Median session duration | Wilcoxon exact | -3.34 (21, 8) |  |  | .000 | .001 |
|  | Consistency of use over time | |  |  |  |  |  |  |
|  |  | % of app minutes in weeks 1-4 | ANOVA |  | -28.9 [-44.5, -13.4] | -3.73 (57) | .000 | .001 |
|  |  | % of app minutes in weeks 5-8 | ANOVA |  | 11.2 [-0.2, 22.6] | 1.97 (57) | .05 | .08 |
|  |  | % of app minutes in weeks 9-12 | ANOVA |  | 17.71 [7.4, 28.0] | 3.43 (57) | .001 | .002 |

*Note*: Wilcoxon = Wilcoxon rank-sum test; Wilcoxon exact = Wilcoxon exact test; *Z* = *Z*-score from the Wilcoxon rank-sum test; LSMeans Diff. = Difference in Least Squares Means; FDR *P* = *P* -value adjusted for false discovery rate; IQR = inter-quartile range; min = minutes; log-min = log-transformed number of minutes; sec = seconds; ERP = exposure with response prevention. For Wilcoxon tests examining pairwise differences against Cluster 3 (n=8), the Wilcoxon exact test with *P* >= |S-Mean| are reported, for cluster comparisons between Clusters 1 and 2, Wilcoxon rank-sum *P* > |Z| is reported.
