# Supplementary material for: Characterizing Behavioral Engagement in Smartphone Cognitive Behavioral Therapy for Obsessive Compulsive Disorder: Secondary Analysis of a Randomized Controlled Trial
Source: JMIR Form Res. 2026 Oct 19;10:e98129. doi: 10.2196/98129 (PMC13679862; doi:10.2196/98129)
Supplement: Supplementary file 6 [file formative-v10-e98129-s006.docx]

**Multimedia Appendix 6**

**Table S1**. Statistical details for the analysis of cluster differences in participant-reported treatment engagement, credibility, expectancy, and satisfaction reported in Table 2.

| **App use variables** | | | **Test** | ***Z* statistic (*n_1_, n_2_*)** | **LSMeans Diff [95% CI]** | ***t*** | **raw *P*** | **FDR *P*** |
| --- | --- | --- | --- | --- | --- | --- | --- | --- |
| ***Comparison of Cluster 1 vs. Cluster 2*** | | | |  |  |  |  |  |
|  | Self-reported treatment engagement | | |  |  |  |  |  |
|  |  | Estimated app usage time, minutes | Wilcoxon | 2.57 (18, 20) |  |  | .01 | .09 |
|  |  | Estimated skill usage time outside the app, minutes | Wilcoxon | 0.76 (18, 20) |  |  | .45 | .56 |
|  | Credibility and Expectancy (CEQ) | |  |  |  |  |  |  |
|  |  | CEQ credibility scores | ANOVA |  | -1.9 [-4.0, 0.1] | -1.90 (57) | .06 | .18 |
|  |  | CEQ expectancy scores | ANOVA |  | -1.3 [-4.0, 1.4] | -0.95 (57) | .35 | .49 |
|  | Satisfaction | |  |  |  |  |  |  |
|  |  | CSQ-8 total scores | ANOVA |  | -1.1 [-3.9, 1.8] | -0.75 (50) | .46 | .56 |
|  |  | uMARS - total mean score | ANOVA |  | 0.0 [-0.3, 0.3] | -0.12 (50) | .91 | .91 |
|  |  | uMARS - recommend the app | ANOVA |  | -0.4 [-0.9, 0.2] | -1.33 (50) | .19 | .32 |
| ***Comparison of Cluster 2 vs. Cluster 3*** | | | |  |  |  |  |  |
|  | Self-reported treatment engagement | | |  |  |  |  |  |
|  |  | Estimated app usage time, minutes | Wilcoxon | n/a |  |  | n/a | n/a |
|  |  | Estimated skill usage time outside the app, minutes | Wilcoxon | n/a |  |  | n/a | n/a |
|  | Credibility and Expectancy (CEQ) | |  |  |  |  |  |  |
|  |  | CEQ credibility scores | ANOVA |  | 4.5 [1.6, 7.3] | 3.15 (57) | .003 | .04 |
|  |  | CEQ expectancy scores | ANOVA |  | 4.6 [0.8, 8.4] | 2.45 (57) | .02 | .10 |
|  | Satisfaction | |  |  |  |  |  |  |
|  |  | CSQ-8 total scores | ANOVA |  | 5.6 [0.8, 10.4] | 2.32 (50) | .02 | .10 |
|  |  | uMARS - total mean score | ANOVA |  | -0.1 [-0.5, 0.4] | -0.40 (50) | .69 | .74 |
|  |  | uMARS - recommend the app | ANOVA |  | 0.9 [-0.0, 1.8] | 1.94 (50) | .06 | .18 |
| ***Comparison of Cluster 1 vs. Cluster 3*** | | | |  |  |  |  |  |
|  | Self-reported treatment engagement | | |  |  |  |  |  |
|  |  | Estimated app usage time, minutes | Wilcoxon | n/a |  |  | n/a | n/a |
|  |  | Estimated skill usage time outside the app, minutes | Wilcoxon | n/a |  |  | n/a | n/a |
|  | Credibility and Expectancy (CEQ) | |  |  |  |  |  |  |
|  |  | CEQ credibility scores | ANOVA |  | 2.6 [-0.4, 5.5] | 1.71 (57) | .09 | .18 |
|  |  | CEQ expectancy scores | ANOVA |  | 3.4 [-0.6, 7.3] | 1.69 (57) | .10 | .18 |
|  | Satisfaction | |  |  |  |  |  |  |
|  |  | CSQ-8 total scores | ANOVA |  | 4.5 [-0.4, 9.4] | 1.84 (50) | .07 | .18 |
|  |  | uMARS - total mean score | ANOVA |  | -0.1 [-0.6, 0.4] | -0.46 (50) | .65 | .73 |
|  |  | uMARS - recommend the app | ANOVA |  | 0.5 [-0.4, 1.4] | 1.12 (50) | .27 | .42 |

*Note*: Wilcoxon = Wilcoxon rank-sum test; Z = z-score from the Wilcoxon rank-sum test; ANOVA = Analysis of Variance; LSMeans Diff = Difference in Least Squares Means; t = test statistic from the pairwise comparison of groups in the ANOVA; DF = degrees of freedom; FDR *P* = *P* -values adjusted for false discovery rate; CEQ = Credibility and Expectancy Questionnaire; CSQ-8 = Client Satisfaction Questionnaire; uMARS = Mobile Application Rating Scale, end-user version.
