# Supplementary material for: Characterizing Behavioral Engagement in Smartphone Cognitive Behavioral Therapy for Obsessive Compulsive Disorder: Secondary Analysis of a Randomized Controlled Trial
Source: JMIR Form Res. 2026 Oct 19;10:e98129. doi: 10.2196/98129 (PMC13679862; doi:10.2196/98129)
Supplement: Supplementary file 7 [file formative-v10-e98129-s007.docx]

**Multimedia Appendix 7**

**Table S1**. Overall (whole sample) app use characteristics of participants (n=60) in a trial studying the efficacy of a 12-week smartphone cognitive behavioral therapy (CBT) program for obsessive compulsive disorder.

|  |  | **Overall**  **(n=60)** | |
| --- | --- | --- | --- |
| **App use variables** | | **M/Md** | **(SD)/[IQR]** |
| **Amount (volume) of use** | |  |  |
|  | Total app use, minutes, Md[IQR] | 278.8 | [189.0, 628.2] |
|  | Total number of app sessions, M(SD) | 59.0 | (33.5) |
|  | Total number of days of app use, M(SD) | 36.9 | (16.8) |
| **Frequency and inactivity periods** | | | |
|  | App use frequency (inactivity), log-minutes, M(SD) | 7.7 | (0.6) |
|  | Number of inactivity periods of 7+ days, Md[IQR] | 1.0 | [0.0, 2.0] |
| **Use of key app features** | |  |  |
|  | % of main modules completed, Md[IQR] | 62.5 | [50.0, 93.8] |
|  | % of main exercises completed, Md[IQR] | 75.0 | [59.7, 96.5] |
|  | % of all exercises (started) completed, Md[IQR] | 93.9 | [86.0, 98.8] |
|  | % of ERP exercises (started) completed, Md[IQR] | 89.7 | [72.1, 100.0] |
|  | Number of ERP exercises completed, Md[IQR] | 9.5 | [4.0, 16.0] |
|  | Number of custom ERP exercises created, Md[IQR] | 0.0 | [0.0, 0.0] |
|  | Number messages sent to coach, M(SD) | 13.1 | (9.6) |
| **Duration of sessions** | |  |  |
|  | % of sessions ≥10 seconds to 3 minutes, M(SD) | 54.8 | (15.2) |
|  | % of sessions >3 minutes to 5 minutes, M(SD) | 9.5 | (5.0) |
|  | % of sessions >5 minutes to 10 minutes, M(SD) | 12.5 | (5.8) |
|  | % of sessions >10 minutes, M(SD) | 23.2 | (14.3) |
|  | Entropy of session duration, Md[IQR] | 1.6 | [1.4, 1.7] |
|  | Median session duration, minutes, Md[IQR] | 2.3 | [1.8, 3.6] |
| **Consistency of use over time** | |  |  |
|  | % of app minutes in weeks 1-4, M(SD) | 59.0 | (20.6) |
|  | % of app minutes in weeks 5-8, M(SD) | 23.8 | (13.9) |
|  | % of app minutes in weeks 9-12, M(SD) | 17.2 | (13.7) |

Note. IQR = inter-quartile range; ERP = exposure with response prevention.
