# Supplementary material for: Characterizing Behavioral Engagement in Smartphone Cognitive Behavioral Therapy for Obsessive Compulsive Disorder: Secondary Analysis of a Randomized Controlled Trial
Source: JMIR Form Res. 2026 Oct 19;10:e98129. doi: 10.2196/98129 (PMC13679862; doi:10.2196/98129)
Supplement: Supplementary file 8 [file formative-v10-e98129-s008.docx]

**Multimedia Appendix 8**

**Table S1**. Overall (whole sample, n=60) participant-reported treatment engagement, credibility, expectancy, and satisfaction reported in Table 2.

|  |  | **Overall** | | |
| --- | --- | --- | --- | --- |
|  |  | **M/Md** | **(SD)/[IQR]** | **n** |
| **Treatment utilization** | |  |  |  |
|  | Estimated app use time, min., Md[IQR] | 306.0 | [144.0, 473.4] | 41 |
|  | Estimated skill use time outside app, min., Md[IQR] | 504.0 | [300.0, 1050.0] | 41 |
| **Credibility and Expectancy (CEQ)** | | | |  |
|  | Credibility, M(SD) | 19.1 | (3.9) | 60 |
|  | Expectancy, M(SD) | 16.4 | (4.9) | 60 |
| **Satisfaction** | |  |  |  |
|  | CSQ-8 total, M(SD) | 25.6 | (5.1) | 53 |
|  | uMARS total, M(SD) | 4.1 | (0.5) | 53 |
|  | uMARS recommend app, M(SD) | 3.8 | (0.9) | 53 |

*Note*. M = mean; md = median; SD = standard deviation; IQR = inter quartile range; min. = minutes; CEQ = Credibility Expectancy Questionnaire; CSQ-8 = Client Satisfaction Questionnaire – 8; uMARS = Mobile Application Rating Scale user version.
